# Acute lung injury in patients with COVID‐19 infection

**DOI:** 10.1002/ctm2.16

**Published:** 2020-03-31

**Authors:** Liyang Li, Qihong Huang, Diane C. Wang, David H. Ingbar, Xiangdong Wang

**Affiliations:** ^1^ Zhongshan Hospital Institute of Clinical Science Shanghai Medical School Fudan University Shanghai China; ^2^ Department of Emergency Sunshine Coast University Hospital Birtinya Queensland Australia; ^3^ Pulmonary, Allergy, Critical Care & Sleep Division, Center for Lung Science and Health University of Minnesota Minnesota USA; ^4^ Jinshan Hospital Center for Tumor Diagnosis & Therapy, Jinshan Hospital Shanghai Medical School Fudan University Shanghai China; ^5^ Shanghai Engineering Research Center of AI Technology for Cardiopulmonary Diseases Shanghai China; ^6^ Shanghai Institute of Clinical Bioinformatics Shanghai China

**Keywords:** ARDS, COVID‐19, lung edema

## Abstract

During the 2020 Spring Festival in China, the outbreak of a novel coronavirus, named COVID‐19 by WHO, brought on a worldwide panic. According to the clinical data of infected patients, radiologic evidence of lung edema is common and deserves clinical attention. Lung edema is a manifestation of acute lung injury (ALI) and may progress to hypoxemia and potentially acute respiratory distress syndrome (ARDS). Patients diagnosed with ARDS have poorer prognosis and potentially higher mortality. Although no effective treatment is formally approved for COVID‐19 infection, support of ventilation with oxygen therapy and sometimes mechanical ventilation is often required. Treatment with systemic and/or local glucocorticoids might be helpful to alleviate the pulmonary inflammation and edema, which may decrease the development and/or consequences of ARDS. In this article, we focus on the lung edema and ALI of patients with this widely transmitted COVID‐19 infection in order to provide clinical indications and potential therapeutic targets for clinicians and researchers.

A novel coronavirus emerged in December 2019, called COVID‐19, with a large number of patients in China.^1^, ^2^ By March 6, 2020, there were a total of 80 710 COVID‐19 cases confirmed, 482 suspected, 53 813 cured, 5737 in intensive care units, and 3045 dead in China, in addition to 17 665 confirmed, 1761 cured, and 343 dead internationally. The World Health Organization designated this as a pandemic on March 11, 2020. The mortality rate of patients with COVID‐19 is estimated to be 0.2‐4.0%, dependent upon therapeutic efficiency and efficacy, locations, and severities. The frequency of asymptomatic or mildly symptomatic COVID‐19 infection is still being determined, so the current mortality rates may be overestimated.

The present article focused specially on the development of acute lung injury (ALI) and acute respiratory distress syndrome (ARDS) in patients with COVID‐19 infection, comparing the difference clinical phenotypes and therapies between COVID‐19‐infected patients inside and outside of the intensive care unit (ICU), and discussing potential alternatives to improve the outcome of COVID‐19‐infected patients with ALI/ARDS. We reviewed 3375 patients with COVID‐19 infection reported in 72 publications (Table 2). Of these infected patients, 342 patients (10.1%) were treated in the ICU and 1278 (37.9%) patients in regular wards (non‐ICU), but the location of care was not clear in some of the patients reported (Table 1).

**TABLE 1:** Clinical characteristics of the patients with COVID‐19 infection reported in the recent studies^*^

	All patients	Patients with ICU	Patients without ICU
Clinical Characteristics	(N = 3375)	(N = 342)^†^	(N = 1278)^†^
Sex (Male; N; %)	1853; 55%	210; 61%	725; 60%
Age (years; mean)	48.67	56.5	45.54
**Clinical symptoms**			
Cough (N; %)	1858; 55%	237; 69%	814; 64%
Expectoration (N; %)	608; 18%	94; 27%	359; 28%
Rhinorrhea (N; %)	17; 0.5%	1; 0.3%	0; 0%
Chest tightness (N; %)	52; 2%	13; 4%	4; 0.3%
Dyspnea (N; %)	522; 15%	122; 36%	195; 15%
Fever (N; %)	2493; 74%	311; 91%	1120; 88%
Fatigue (N; %)	956; 28%	162; 47%	504; 39%
Chill (N; %)	177; 5%	29; 8%	105; 8%
Anorexia (N; %)	95; 3%	34; 10%	43; 3%
Respiratory rate (mean)	20.38 (N = 363)	20.94 (N = 34)	20 (N = 197)
Heart Rate (mean)	88.37 (N = 510)	85.72 (N = 35)	86.93 (N = 197)
Systolic/diastolic pressure(mean)	126.10/79.60 (N = 413)	134.59/79.63 (N = 48)	122.23/76.94 (N = 123)
**Clinical examinations**			
Leukocytes (3.5‐9.5)	4.93 (N = 2161)	5.12 (N = 329)	4.84 (N = 1241)
Neutrophils (1.8‐6.3)	3.61 (N = 822)	6.16 (N = 84)	2.98 (N = 225)
Lymphocytes (1.1‐3.2)	1.17 (N = 2155)	0.75 (N = 329)	1.00 (N = 1241)
Monocytes (0.1‐0.6)	0.42 (N = 352)	0.39 (N = 45)	0.39 (N = 142)
CRP (0‐10)	24.63 (N = 735)	53.46 (N = 107)	18.96 (N = 185)
PCT (0‐0.05)	0.32 (N = 571)	0.13 (N = 94)	0.06 (N = 150)
D‐Dimer (0‐0.5)	0.70 (N = 935)	2.38 (N = 118)	0.3 (N = 205)
LDH (125‐243)	262.20 (N = 767)	414.65 (N = 86)	228.72 (N = 233)
ESR (0‐15)	35.93 (N = 235)	75.25 (N = 9)	41.23 (N = 40)
CD3+ (955‐2860)	529.33 (N = 52)	314.34 (N = 9)	621.81 (N = 40)
CD4+ (450‐1440)	263.70 (N = 61)	221.32 (N = 9)	352.75 (N = 40)
CD8+ (320‐1250)	149.09 (N = 61)	145.35 (N = 9)	201 (N = 40)
CD19+ (90‐560)	101.65 (N = 52)	88.32 (N = 9)	109.31 (N = 40)
CD16+CD56+ (150‐1100)	102.80 (N = 52)	110.6 (N = 9)	69.79 (N = 40)
SpO2 (94‐100)	96.10 (N = 466)	92.03 (N = 4)	97.07 (N = 63)
PaO2 (75‐100)	89.60 (N = 63)	68.00 (N = 36)	‐
PaO2/FiO2 (400‐500)	191.42 (N = 23)	133/58 (N = 48)	‐
**Imaging manifestation**			
Ground glass/patchy shadows (N; %)	1984 (N = 2246); 88%	249 (N = 304); 82%	951 (N = 1105); 86%
Consolidation (N; %)	341 (N = 972); 35%	19 (N = 304); 6%	21 (N = 1105); 2%
Air bronchogram (N; %)	236 (N = 661); 36%	8 (N = 304); 3%	15 (N = 1105); 1%
Pleural effusion (N; %)	32 (N = 532); 60%	2 (N = 304); 1%	2 (N = 1105); 0.2%
Lobes involved (mean)	2.95 (N = 634)	4.67 (N = 22)	2.61 (N = 77)
Bilateral pneumonia (N; %)	1383 (N = 2190); 63%	241 (N = 245); 98%	565 (N = 1138); 50%
Unilateral pneumonia (N; %)	126 (N = 2190); 58%	4 (N = 245); 2%	568 (N = 1138); 50%
**Treatment (N; %)**	N = 2145	N = 260	N = 1111
Antiviral therapy	1359; 63%	166; 64%	495; 45%
IFN inhalation	231; 11%	32; 12%	11; 1%
Antibiotic therapy	1256; 59%	178; 68%	563; 51%
Antifungal therapy	46; 2%	13; 5%	18; 2%
Corticosteroids	424; 20%	126; 48%	175; 16%
Immunoglobulin treatment	319; 15%	89; 34%	87; 8%
Oxygen therapy	937; 44%	163; 63%	478; 43%
Noninvasive ventilation	173; 8%	83; 32%	2; 0.2%
Invasive mechanical ventilation	64; 3%	55; 21%	0; 0%
ECMO	16; 1%	13; 5%	0; 0%
**Prognosis (N; %)**	N = 2442	N = 203	N = 969
Death	83; 3%	21; 10%	2; 0.2%
Discharged	521; 21%	17; 8%	81; 8%
Hospitalization	1838; 75%	165; 81%	886; 91%

* The patients were summarized on the basis of the clinical characteristics from the recent studies listed in Table 2.

† We summarized 3375 patients with COVID‐19 infection, among whom 342 patients with ICU care and 1278 patients without ICU care had detailed clinical data. Other 1755 patients were not concluded in the subgroup analysis due to the lack of clinical data in the original study.

**Abbreviation**: CRP, C‐reactive protein; PCT, procalcitonin; ESR, erythrocyte sedimentation rate; LDH, lactate dehydrogenase; IFN, interferon; ECMO, extracorporeal membrane oxygenation.

**TABLE 2:** The references reviewed in the article

	Author	Journal	Volume/DOI/access website
1	Xiang, et al	Chinese J of Resp Crit Care	http://kns.cnki.net/kcms/detail/51.1631.r.20200228.1506.002.html.nonbreakingspace
2	Chen, et al	The Lancet	2020; 395(10223): 507–513
3	Chan, et al	The Lancet	2020; 395(10223): 514–523
4	Huang, et al	The Lancet	2020; 395(10223): 497–506
5	Zhu et al	N Engl J Med	2020; 382(8): 727–733
6	Lei, et al	Radiology	10.1148/radiol.2020200236.
7	Holshue, et al	N Engl J Med	10.1056/NEJMoa2001191.
8	He, et al	Chin J Integr Trad West Med	10.7661/j.cjim.20200216.276.
9	Liu, et al	Chin J Diffic and Compl Cas	http://kns.cnki.net/kcms/detail/13.1316.R.20200302.1016.002.html.
10	Huang, et al	J Emerg Tradit Chin Med	2020; 29(3): 381–384
11	Cao, et al	Med J of Wuhan Univ	10.14188/j.1671‐8852.2020.0087.
12	Zhao, et al	Modern Oncology	2020; 28(8): 1–4
13	Zhou, et al	Shanghai J Prev Med	10.19428/j.cnki.sjpm.2020.20078.
14	Zou, et al	Medical Journal of Wuhan University	10.14188/j.1671‐8852.2020.0095.
15	Ruan, et al	Shanghai J Tradit Chin Med	2020; 54(4):14‐17
16	Zhang, et al	Chin J Zoonoses	http://kns.cnki.net/kcms/detail/35.1284.r.20200225.2006.002.html.
17	Chen, et al	Chin J Clin Med	2020;27(1): 1–4
18	Fang, et al	Chin Pharmacol Bulletin	2020; 36(4): 12–18
19	Liu, et al	J Jilin Univ (Med Edition)	2020; 46(2): 410–414
20	Hu, et al	Chin J Resp Crit Care Med	2020; 19(2): 1–4
21	Liu, et al	Med J Wuhan Univ	10.14188/j.1671‐8852.2020.0078.
22	Ling, et al	Prev Med	2020; 32(02): 109–112
23	Wang, et al	Med J Wuhan Univ	10.14188/j.1671‐8852.2020.0080.
24	Yang, et al	Chin J Med Imaging Technol	2020; 36(2): 314–315
25	Ji, et al	Chin J Med Imaging Technol	2020; 36(2): 242–247
26	Liu, et al	Chin J Diffic and Compl Cas	2020; 19(2): 190–191
27	Shen, et al	J Dalian Med Univ	2020; 42(1): 32–36
28	Liu, et al	Radiologic Practice	10.13609/j.cnki.1000‐0313.2020.03.001.
29	Gong, et al	Radiologic Practice	10.13609/j.cnki.1000‐0313.2020.03.002.
30	Pan, et al	Chongqing Medicine	http://kns.cnki.net/kcms/detail/50.1097.R.20200215.2009.002.html.
31	Yu, et al	Beijing J Tradit Chin Med	http://kns.cnki.net/kcms/detail/11.5635.R.20200215.2008.002.html.
32	Han, et al	Chin J Clin Thoracic and Cardiovascular Surgery	2020; 27(4): 1–3
33	Kong, et al	Chin J Clin Med	http://kns.cnki.net/kcms/detail/11.5635.R.20200215.2008.002.html.
34	Chung, et al	Radiology	10.1148/radiol.2020200230.
35	Yang, et al	J Infect	10.1016/j.jinf.2020.02.016.
36	Zhang, et al	Virol Sin	10.1007/s12250‐020‐00203‐8.
37	Wang, et al	Biosci Trends	10.5582/bst.2020.01030.
38	Liu, et al	Sci China Life Sci	10.1007/s11427‐020‐1643‐8.
39	Bastola, et al	Lancet Infect Dis	2020; 20(3): 279–280
40	Pan, et al	Eur Radiol	10.1007/s00330‐020‐06731‐x.
41	Silverstein, et al	Lancet	2020; 395(10225): 734
42	Xu, et al	Eur J Nucl Med Mol Imaging	10.1007/s00259‐020‐04720‐2.
43	Fang, et al	QJM	10.1093/qjmed/hcaa038.
44	Tang, et al	J Thromb Haemost	10.1111/jth.14768.
45	Hao W	J Infect	10.1016/j.jinf.2020.02.008.
46	Wu, et al	Invest Radiol	10.1097/RLI.0000000000000670.
47	Hao W, et al	Clin Microbiol Infect	10.1016/j.cmi.2020.02.011.
48	van Cuong, et al	Lancet Infect Dis	10.1016/S1473‐3099(20)30111‐0.
49	Xu, et al	Lancet Respir Med	10.1016/S2213‐2600(20)30076‐X.
50	Wei, et al	Korean J Radiol	10.3348/kjr.2020.0112.
51	Xu, et al	Eur J Nucl Med Mol Imaging	10.1007/s00259‐020‐04735‐9.
52	Xu, et al	BMJ	2020; 368: m606
53	Xu, et al	J Infect	10.1016/j.jinf.2020.02.017.
54	Wu, et al	Clin Infect Dis	10.1093/cid/ciaa199.
55	Guan, et al	N Engl J Med	10.1056/NEJMoa2002032.
56	Huang, et al	J Microbiol Immunol Infect	10.1016/j.jmii.2020.02.009.
57	Cai, et al	Clin Infect Dis	10.1093/cid/ciaa198.
58	Tian, et al	J Infect	10.1016/j.jinf.2020.02.018.
59	Liu, et al	Chin J Tubere Respir Dis	2020; 43(00): E016‐E016
60	Wang, et al	JAMA	10.1001/jama.2020.1585.
61	Zhang, et al	Allergy	10.1111/all.14238.
62	Liu, et al	Chin Med J (Engl)	10.1097/CM9.0000000000000744.
63	Han, et al	J Med Virol	10.1002/jmv.25711.
64	Zhang, et al	Chin J Pediatr	2020; 58(3): 182–184
65	Cai, et al	Chin J Pediatr	2020; 58(2): 86–87
66	Chen, et al	Chin J Pediatr	10.3760/cma.j.issn.0578‐1310.2020.03.000.
67	ZengL, et al	Chin J Pediatr	2020; 58(00): E009‐E009
68	Kim, et al	J Korean Med Sci	2020; 35(5): e61
69	Duan, et al	Radiology	10.1148/radiol.2020200323.
70	Song, et al	Radiology	10.1148/radiol.2020200274.
71	Fang, et al	Radiology	10.1148/radiol.2020200280.
72	Shi, et al	Radiology	10.1148/radiol.2020200269.

Lung edema is one of the clinical characteristics and critical stages of patients with severe COVID‐19 infections. Clinical phenotypes of patients with COVID‐19 infection‐induced lung edema were summarized from a large population of patients collected from 80 reports.^3^ Although chest radiographies are nonspecific/nondiagnostic, acute lung edema and injury were evidenced in patients with COVID‐19 infection by multiple opacities and consolidations with or without air‐bronchogram mainly distributed in peripheral lung lesions.^4^ Patients with milder illness only had ground‐glass opacities, indicating less edematous fluid and often improved with time and treatment.^1^ With greater severity or with disease progression, the density and distribution of opacities and bilateral lobular and subsegmental lesions of consolidation increased.^5^

The occurrence and severity of ALI are a major determining factor of the prognosis of patients with COVID‐19 infection. About 30% patients with COVID‐19 infection in ICU developed severe lung edema, dyspnea, hypoxemia, or even ARDS. Another study reported that 17/99 (17%) patients (including ICU and non‐ICU patients) with COVID‐19 infection developed ARDS and about 65% (11/17) of patients with ARDS died,^6^ indicating that patients with ARDS had worse prognosis. Autopsy and radiographic findings in patients with COVID‐19 infection demonstrated acute lung inflammation characterized by leukocyte infiltrations, lung endothelial barrier dysfunction by tissue edema, and tissue injury by alveolar wall damage.^7^, ^8^ One report described chest CT findings in 81 patients and noted that characteristically the findings were bilateral (79%), peripheral (54%), ill‐defined (81%), and ground glass (65%).^9^ In the compiled data (Table 1), the chest CT manifestations observed in patients requiring ICU care showed more shadows with consolidation (6% vs 2%) and air bronchograms (3% vs 1%) than those not requiring ICU care. In addition, almost all the ICU patients had bilateral chest radiographic abnormalities (pneumonia, pulmonary edema, and/or ARDS), while about half of the patients outside of ICU had unilateral pneumonia.

Infection is one of the major etiologies that can induce the lung edema of ARDS, although the exact mechanisms may vary among pathogens. Neutrophil activation is a long‐recognized amplifier of lung injury in ARDS. Given the rapid onset of the COVID‐19 pandemic, relatively little is known about the pathophysiology of the lung injury. Most relevant data derived from other viral illnesses, such as influenza or other corona viral infections (MERS and SARS). At present it is uncertain whether COVID‐19 has distinct or unique pathophysiologic mechanisms compared to other viral lung injuries. Recent data on influenza infection found that neutrophil extracellular traps play an important role in pathogenesis of lung inflammation to contribute to ALI.^10^ In animal models of influenza infection, excessive neutrophilic infiltration in the lung is an important step in development of ARDS‐like pathological alterations, including lung inflammation, lung edema, hypoxemia, and diffuse alveolar damage.^10^ The severity of lung injury in these models is ameliorated by the depletion of neutrophils.^11^ Similarly, in human clinical studies the severity of influenza lung damage was heterogeneous and associated with the neutrophil‐associated and interferon‐induced responses in individual patients.^11^ In COVID‐19 patients changes of circulating neutrophil count was associated with the severity of the disease; for example, patients with secondary bacterial infections in ICU had higher levels of neutrophils than those cared outside of the ICU.^1^, ^6^ Table 1 demonstrated that levels of white blood cells and neutrophils in the 329 ICU patients with COVID‐19 were higher than those in the 1241 non‐ICU patients, while levels of lymphocytes in patients in ICU were lower.

As a syndrome triggered by many specific causes, the clinical and pathological development of ALI/ARDS has common pathophysiologic mechanisms and features, although initial risk factors and causes may vary. It is generally presumed that COVID‐19 infections may induce diffuse alveolar damage, overproduction of inflammatory factors, and increase vascular permeability, thereby causing progressive hypoxemia. There are a small number of reports of lung pathology in COVID‐19 infections that support this presumption, showing diffuse alveolar damage, desquamated type II alveolar epithelial cells, hyaline membranes, and fibro myxoid exudates.^8^ Patients with COVID‐19 infection who required ICU care had greater clinical symptomatology, including dyspnea, fatigue, and anorexia, as well as the common symptoms of fever and cough, as detailed in Table 1. Low levels of oxygen saturation demonstrated the existence of hypoxemia in ICU patients with COVID‐19 infection.

Corona viruses interact with respiratory epithelium based upon the binding of corona spike proteins to specific cell surface protein and/or sugar receptor molecules.^12^, ^13^ The binding proclivities of the specific spike proteins explain some of the different behaviors of the SARS and MERS corona virus infections. The COVID‐19 spike protein can bind the angiotensin 2 converting enzyme 2 (ACE2) receptors in both the lung and gastrointestinal tract. While the COVID‐19 receptor binding domain (RBD) significantly differs from SARS‐CoV RBD, especially in two regions when binding to ACE2,^14^ the spike proteins of both viruses can bind ACE2. Stimulation of ACE2 receptors can dilate blood vessels, alleviate inflammation, and reduce oxidation stress in the cardiovascular system.^15^ Experimental evidence suggests that ACE2 might play a decisive role in the regulation of the amount of edema in lung alveolar during the development of ARDS.^16^ SARS‐CoV can interact with ACE2 and reduces ACE2 expression in human cells, leading to lung edema.^17^, ^18^ It is possible that COVID‐19 may cause lung edema and ALI/ARDS through binding to ACE2.

Efficient prevention and treatment of lung edema and ALI/ARDS are critical in optimizing the prognosis of ICU patients with COVID‐19 infection. A number of potential therapies are under development or undergoing clinical trials for COVID‐19.^19^ Antiviral therapy may be an important primary treatment in patients with COVID‐19 infection, along with respiratory and oxygen support. Compared with non‐ICU patients, ICU patients received more therapeutically directed medications, especially more inhaled interferon (IFN)‐α‐2b, corticosteroids, and immunoglobulin treatment (Table 1). In experimental studies, there are similar pathophysiology and pro‐inflammatory mediators involved in different severe viral pneumonias. However, there may be unique features to specific viruses, including COVID‐19. The neutralization of IFN‐γ may be an alternative treatment strategy for COVID‐19‐associated ALI/ARDS, since serum levels of IFN‐γ are higher in patients with COVID‐19 infection. The recombinant ACE2 protein may be another potential therapeutic candidate to protect against ALI/ARDS patients, as indicated in patients with H5N1, H7N9, or SARS infections.^17^, ^20^, ^21^ However, no significant improvement in clinical outcomes of ARDS patients was found in a clinical small‐size phase II study where severe participants were enrolled in the study.^22^ It may be worthwhile to explore the therapeutic effects of recombinant ACE2 protein in the early stage of lung edema or COVID‐19 infection.

The maintenance of open airways and improvement of hypoxemia are necessary approaches to prevent and treat ALI/ARDS in patients with COVID‐19 infection. The correction of hypoxemia is an essential component in treatment of lung edema and ALI. Current data show that half of all patients with COVID‐19 infection required oxygen therapy, including nasal catheter oxygen inhalation, invasive, or non‐invasive mechanical ventilation, or extracorporeal membrane oxygenation (ECMO) (Table 1). Of course, ICU patients more often needed mechanical ventilation, especially noninvasive ventilation, invasive mechanical ventilation, and ECMO due to severe hypoxemia. Based on dynamic altered alveolar mechanics of alveolar sizes and shapes in ALI and ARDS, use of lung protective ventilation strategy with tidal volume at 4–8 mL/kg predicted body weight is recommended to prevent further lung injury.^23^ The time‐controlled adaptive ventilation protocol was proposed as the primary mode of mechanical ventilation in COVID‐19‐infected patients with high risk of ARDS, meaning an extended time of inspiration and brief time of expiration to prevent alveolar collapse at the end of expiration.^24^ The incidence of ALI/ARDS has been improved with development of supportive technologies and approaches, while the mortality is 6% of the 342 ICU patients with COVID‐19 infection, as compared with 0.15% of the 1278 non‐ICU patients, as shown in Table 1.

Systemic corticosteroids may alleviate the inflammation and lung edema, but ideally should be used for as short duration of treatment as possible.^25^ Clinical studies demonstrated that corticosteroid was applied to 28 (20%) patients with severe COVID‐19 infection, for example, methylprednisolone at 40–80 mg daily for 5 days on average (IQR: 3–8 days).^1^, ^6^ Patients with more severe infection or ICU care were more often treated with corticosteroid than the non‐ICU patients (49% vs 16%), although the use of corticosteroid in COVID‐19 infection is still controversial since there is not a strong evidence base for this practice.

There is still an urgent need for specific therapy for ALI/ARDS, since almost all promising candidates have failed to prove beneficial in clinical trials.^25^ For example, Cisatracurium as one of neuromuscular blockers failed to improve the prognosis of patients with ARDS in several clinical trials.^26^ Other treatments including mesenchymal stem cell transplantation are still under study.^27^, ^28^

The lung inflammation and edema are a key process of the development from ALI to ARDS as well as multiple organ dysfunction/failure syndrome. The interaction between COVID‐19 and alveolar epithelial cells may play the decisive role in the development of the gas‐blood barrier dysfunction, since activated epithelial cells can act as the primary receiver of pathogens and the initiator of secondary inflammation locally and systemically.^29^ The activated epithelial and endothelial cells overproduce pro‐inflammatory cytokines such as IL‐1, IL‐6, and IL‐17A to initiate the inflammatory response leading to ALI.^30^ The C‐reactive protein (CRP), procalcitonin (PCT), D‐dimer, erythrocyte sedimentation rate (ESR), and lactate dehydrogenase (LDH) were elevated in ICU patients with COVID‐19 infection, as compared to non‐ICU patients (Table 1). Those clinical indices related to inflammation may provide track with the severity of the disease. The impairment of the alveolar‐capillary barrier and the accumulation of edematous fluid containing numerous pro‐inflammatory mediators in the alveolar can lead to the occurrence of an inflammatory storm.^31^ ALI/ARDS may occur in the early stage of systemic inflammation syndrome and be the first organ dysfunction in the development of multiple organ dysfunction syndrome.

ALI/ARDS is a central component of the pathophysiological processes by which patients with severe COVID‐19 infection proceed to develop multiple organ dysfunction with high mortality.^32^ The median time of ARDS occurrence was about 9 days from the onset of severe COVID‐19 infection^1^ and patients with ARDS died a mean of 20 days after the onset of the symptoms or about 9–11 days after ICU admission.^33^ The clinical status of COVID‐19 patients without ARDS improved approximately 8 days after hospital treatment.^34^ Thus, the early treatment of lung edema and ALI is important to control the progression of COVID‐19 infection and improve the prognosis of patients with ARDS in clinical management.

## CONFLICT OF INTEREST

The authors declare no conflict of interest.
